# Hospital-Associated Multidrug-Resistant MRSA Lineages Are Trophic to the Ocular Surface and Cause Severe Microbial Keratitis

**DOI:** 10.3389/fpubh.2020.00204

**Published:** 2020-06-03

**Authors:** Paulo J. M. Bispo, Lawson Ung, James Chodosh, Michael S. Gilmore

**Affiliations:** ^1^Department of Ophthalmology, Massachusetts Eye and Ear, Harvard Medical School, Boston, MA, United States; ^2^Infectious Disease Institute, Harvard Medical School, Boston, MA, United States; ^3^Department of Microbiology and Immunobiology, Harvard Medical School, Boston, MA, United States

**Keywords:** MRSA, Ocular infection, Molecular Epidemiology, Tissue tropism, biogeography of infections

## Abstract

Methicillin-resistant *Staphylococcus aureus* (MRSA) is a common cause of severe and difficult to treat ocular infection. In this study, the population structure of 68 ocular MRSA isolates collected at Massachusetts Eye and Ear between January 2014 and June 2016 was assessed. By using a combination of multilocus sequence typing (MLST) analysis, SCC*mec* typing and detection of the panton-valentine leukocidin (PVL) gene, we found that the population structure of ocular MRSA is composed of lineages with community and hospital origins. As determined by eBURST analysis of MLST data, the ocular MRSA population consisted of 14 different sequence types (STs) that grouped within two predominant clonal complexes: CC8 (47.0%) and CC5 (41.2%). Most CC8 strains were ST8, harbored type IV SCC*mec* and were positive for the PVL-toxin (93.7%). The CC5 group was divided between strains carrying SCC*mec* type II (71.4%) and SCC*mec* type IV (28.6%). Remaining isolates grouped in 6 different clonal complexes with 3 isolates in CC6 and the other clonal complexes being represented by a single isolate. Interestingly, major MRSA CC5 and CC8 lineages were isolated from discrete ocular niches. Orbital and preseptal abscess/cellulitis were predominantly caused by CC8-SCC*mec* IV PVL-positive strains. In contrast, infections of the cornea, conjunctiva and lacrimal system were associated with the MDR CC5 lineage, particularly as causes of severe infectious keratitis. This niche specialization of MRSA is consistent with a model where CC8-SCC*mec* IV PVL-positive strains are better adapted to cause infections of the keratinized and soft adnexal eye tissues, whereas MDR CC5 appear to have greater ability in overcoming innate defense mechanisms of the wet epithelium of the ocular surface.

## Introduction

Antimicrobial resistance in human infections has reached alarming levels and has become one of the major public health threats of the twenty first Century (1). Methicillin-resistant *Staphylococcus aureus* (MRSA) remains a leading cause of antibiotic-resistant infections at many anatomical sites (2, 3). MRSA initially were confined to the hospital environment, but in the mid 1990s began to proliferate in the community (4), and are now leading causes of antibiotic-resistant infections in both settings (5). In US, strains within lineages that constitute clonal complex 5 (CC), notably the USA100 clone, are most commonly hospital-associated (5). USA300, a representative of CC8, has emerged as the most prevalent CA-MRSA clone in the US (5, 6). USA300 has also invaded the hospital setting where it is now a common cause of MRSA infections in American hospitals (7).

Ocular infections caused by MRSA have become increasingly common in the last two decades (8–11). These infections have been associated with serious ocular damage and permanent vision loss (12, 13), including bilateral blindness (14). Despite the growing importance of MRSA in ophthalmology, little is known about the population structure of MRSA causing the most common eye infections, or the microbial and host features that dictate this structure. The eye has extensive defenses for protection of vital structures from constant environmental exposure. These include mechanical barriers (e.g., lids, lashes), a polarized wet epithelium, a secreted tear film containing immunoglobulins and various other antimicrobial factors, mucins (secreted MUC5AC and shed epithelial cell surface-associated transmembrane mucins MUC1, MUC4, and MUC16), and cells of the innate immune system (15–17).

This unique environment of the ocular wet mucosa and its components are expected to act as selective forces that can shape the spatial distribution of microorganisms colonizing and infecting this ocular niche. The study of these ecological and geographical forces, as classically applied in ecology to study the biogeography of life in the natural world can now be combined with refined genetic and genomic epidemiology data to advance our understanding of community structures and distribution of microbes in different body sites (18, 19). We previously reported the genomic characteristics of a divergent cluster of unencapsulated *Streptococcus pneumoniae* strains that are uniquely tropic and adapted to the conjunctiva (20). These strains carry a set of genes that are absent or substantially different from those encoded within the genomes of encapsulated respiratory strains, which appear to be important for the pathogenesis of epidemic conjunctivitis. We have demonstrated that a unified model of microbial biogeography that incorporates classic ecological principles to explain community assemblage and dynamics can be applied to the understanding of this radical bifurcation in phylogeny and niche subspecialization of the unencapsulated *S. pneumoniae* conjunctivitis cluster (21). Because MRSA now rank among leading causes of a variety of ocular infections, to gain insight into particular features of importance in the pathogenesis of infection, it was of interest to determine the microscale biogeography of MRSA eye infections, whether dominant genetic lineages were associated with all sites of infection, or if there was evidence of a tissue tropism that would drive a specific population structure. We report that the population structure of ocular MRSA strains isolated at Massachusetts Eye and Ear (MEE) is dominated by the two major clonal complexes that cause infections at other body sites, but exhibit a distinct distribution in the types of infection they cause.

## Methods

### Bacterial Strains

Protocols for obtaining bacterial isolates collected for infection diagnosis were approved by the MEE Institutional Review Board (IRB). Since this study only included discarded bacterial isolates that were frozen in our pathogen repository, written informed consent was waived by the MEE IRB. In total, 68 consecutive MRSA isolates recovered from January 2014 to June 2016 were analyzed for this study. For patients from whom multiple isolates from the same eye were obtained for infection diagnosis within a period of 6 months, only the first isolate was included. Specimens were obtained by the attending ophthalmologist or resident physician following institutional guidelines and submitted to the clinical laboratory for processing. Suspected *S. aureus* colonies were routinely identified using a combination of phenotypic methods including detection of coagulase and protein A by latex agglutination, followed by confirmation of species and antimicrobial susceptibility testing using the MicroScan Walkaway 40 Plus System (Beckman Coulter, Brea, CA). Isolates were stored at −80°C in Microbank™ cryopreservative tubes (ProLab Diagnostics). Frozen isolates were cultured twice on blood agar before further testing.

### Clinical Data Collection and Statistical Analyses

Demographic data and risk factors for MRSA infection were collected using the IRB-approved Research Electronic Data Capture (REDCap) tool, hosted by MEE and Harvard Medical School (22). General demographic data included age, sex and ethnicity. Ocular comorbidities, including any ophthalmic surgical history, ocular surface disease, eyelid disease, lacrimal system dysfunction, atopy, contact lens use and trauma were collected (see **Table 2** legend for full definitions). Patient systemic comorbidities and previous healthcare exposures were also captured. To identify possible healthcare exposures which may potentiate selective pressures for antibiotic-resistant infection, we identified these following groups in our data: patients residing in nursing homes and/or residential facilities; those requiring chronic ambulatory care such as renal replacement therapy (dialysis) and hospital-based infusions; and patients who had either inpatient hospital admission and/or day admission for eye surgery within the preceding 3 prior to developing an MRSA infection. For patients with MRSA keratitis, we recorded the presenting features of the ulcers according to an institution-wide clinical algorithm which mandates the collection of corneal cultures for lesions meeting any of the following criteria: ≥1+ cells in the anterior chamber; ≥2 mm infiltrate and/or the presence of ≥2 satellite lesions; or infiltrate located ≤ 3 mm from the corneal center (23). Simple 2 by 2 tests of proportion (Fischer's exact test) were used to compare CC5 and CC8 groups according to collected categorical variables, while age was compared using the non-parametric Wilcoxon rank-sum test.

### Antimicrobial Susceptibility Testing

*In vitro* susceptibility to ciprofloxacin (Fluka), ofloxacin, levofloxacin (TCI America), moxifloxacin, and besifloxacin (Sigma-Aldrich) was performed by broth microdilution methods according to the Clinical and Laboratory Standards Institute (CLSI) (24). Quality control was performed by testing the *S. aureus* ATCC 29213 control strain. The interpretative criteria for each antimicrobial agent tested were those published by CLSI (25).

### DNA Extraction

DNA extraction was performed using Chelex 100 molecular biology resin (Bio-Rad) as previously described (26). Purified genomic DNA was diluted 1:10, and was assessed for purity and DNA concentration using a Synergy 2 Multi-Mode Plate Reader and Take3 software system (BioTek).

### SCC*mec* Typing

PCR-based genotyping of the chromosomal cassette recombinase (*ccr*) and *mec* complexes comprising the SCC*mec* was determined by a combination of multiplex PCR designed to classify the *mec* complex and *ccr* complex using a previously published protocol (27). For each multiplex PCR assay, reference MRSA strains for SCC*mec* types II (USA100) and IV (USA800), provided by the Network of Antimicrobial Resistance in *Staphylococcus aureus* (NARSA) were included. SCC*mec* was considered nontypeable if *mec* and/or *ccr* complex gave no amplification results, if the isolate carried more than one *ccr* or *mec* complex, or if there was a *mec*/*ccr* complex combination not previously described.

### PVL Detection

The presence or absence of the Panton-Valentine Leukocidin (PVL) toxin gene was determined by PCR amplification of the *LukS-PV-lukF-PV* genes as previously described (28). Reference MRSA strains (provided by NARSA) USA300 and USA100 served as positive and negative controls, respectively.

### MLST

Multilocus sequence typing (MLST) was performed for all MRSA isolates using a scheme based on the sequencing of internal fragments of seven *S. aureus* housekeeping genes (*arcC, aroE, glpF, gmk, pta, tpi*, and *yqiL*). The PCR products were purified (QIAquick PCR purification kit; Qiagen), and both strands were sequenced by Genewiz Incorporated (South Plainfield, NJ). The sequences obtained were edited using Geneious R8 and sequence types (STs) were assigned using the *S. aureus* MLST database (https://pubmlst.org/saureus/). Clonal complexes (CC) were determined using the go eBURST algorithm (http://www.phyloviz.net/goeburst/).

### Statistics

Descriptive statistics were calculated using SPSS software (version 25, IBM, Armonk, New York), and proportions were compared by χ^2^or Fisher exact test, as appropriate. A *P* value of < 0.05 was considered statistically significant.

## Results

A total of 75 MRSA were identified from 281 *S. aureus* recovered from ocular sites at MEE from January 2014 to June 2016 (overall rate of 26.7%). The proportion of ocular MRSA isolates did not change considerably in 2014 (25.9%) compared to 2015 (22.3%), but was substantially higher during the sampling period of 2016 (37.7%). Of those, 7 MRSA were obtained from second cultures of the same patient eye, and were excluded from further study. The remaining 68 non-duplicate MRSA isolates were then analyzed. Sites of infection from which MRSA were isolated included orbital and preseptal abscess/cellulitis (*n* = 27), keratitis (*n* = 14), conjunctivitis (*n* = 9), lacrimal system infection (*n* = 8), eyelid margin infections (*n* = 4), endophthalmitis (*n* = 2), and miscellaneous (*n* = 4).

### Two Major Clonal Complexes Dominate the Ocular MRSA Population

**Figure 1:**
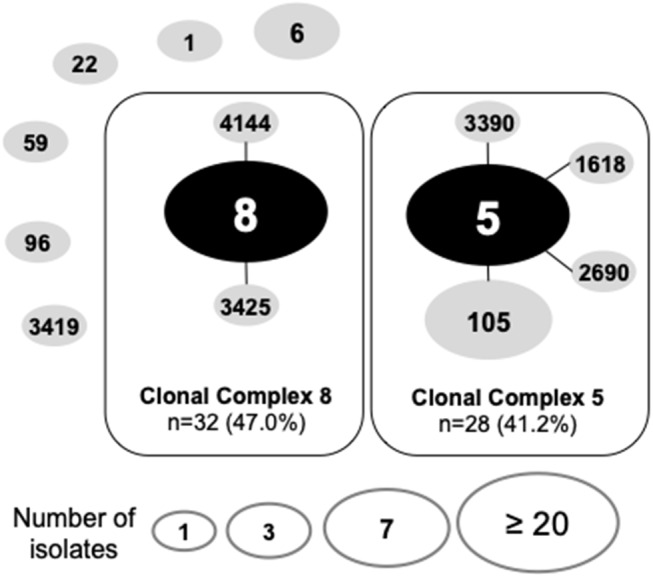
Go eBurst-based population structure of ocular MRSA strains (*n* = 68). Each ST is represented by a circle. Lines connect single-locus variants. The black circles represent clonal complex founders. Circles not connected represent singleton STs in this particular population structure.

Despite the clinical importance of MRSA, much remains to be learned about the pathogenesis of infection at different anatomical sites on and around the eye. Because the tissues of the eye and adnexa differ widely in host defenses (e.g., wet epithelium vs. keratinized epithelium and soft tissues), it was of interest to know whether some MRSA lineages were enriched in pathogenic features that select for one MRSA lineage over another. By using a combination of multilocus sequence typing analysis, SCC*mec* typing and detection of the PVL-toxin encoding gene, we found that the population structure of ocular MRSA is diverse, but dominated by the CC5 and CC8 lineages associated with infection at other anatomical sites (4, 5). As determined by eBURST analysis of MLST data (Figure 1), 14 different sequence types (STs) were identified, with most belonging to clonal clusters CC8 (47.0%, *n* = 32) and CC5 (41.2%, *n* = 28). The clonal cluster CC6 encompassed 3 strains (4.4%), and 5 strains represented single sequence types. Most CC8 strains (93.7%, *n* = 30) were ST8, harbored a SCC*mec* type IV and were positive for the PVL-toxin, common features of the USA300 strain. The CC5 group could be divided into those carrying a SCC*mec* type II (71.4%, *n* = 20), which includes isolates with the characteristics of the USA100 clone, and (28.6%, *n* = 8) SCC*mec* type IV, typical of the USA800 clone (Table 1).

**Table 1:** SCC*mec* typing and PVL detection according to the clonal complex.

**Clonal complex (No.)**	**SCC*****mec*** **typing**		**No. PVL positive**
	**Type**	**No. (% of total)**	
CC8 (32)	IV	31 (45.6)	30
	V	1 (1.5)	1
CC5 (28)	II	20 (29.4)	–
	IV	8 (11.7)	–
CC6 (3)	IV	3 (4.4)	–
CC1 (1)	IV	1 (1.5)	1
CC15 (1)	II	1 (1.5)	1
CC22 (1)	IV	1 (1.5)	1
CC59 (1)	IV	1 (1.5)	1
CC96 (1)	NT	1 (1.5)	–

Age at presentation ranged from 2 to 102 years (median 53.08) with CC5-infected patients being significantly older (median age, 68.05 vs. 35.9, *p* < 0.001) (Table 2). CC5-infected patients were more frequently subjected to eye surgery (66.7 vs. 16.7%, *p* < 0.001), especially cataract surgery with implantation of intraocular lenses (44.4 vs. 10%, *p* = 0.006). Healthcare exposure was more common among patients infected with CC5 strains, including higher rates of topical antibiotic use at presentation (55.6 vs. 10.0%, *p* < 0.001) and prior to presentation (48.5 vs. 16.7%, *p* = 0.02). There was also a higher proportion of patients known to require non-acute clinical care and/or residents of aged care facilities in the CC5 group (22.2 vs. 3.3%, *p* = 0.05).

**Table 2:** Demographic and clinicopathologic data for patients with culture-positive MRSA ocular infections at MEE, 2014–2016 (*n* = 58).^a^

**Patient characteristics**	**MRSA clonal group**		***P* value**
	**CC5 (*n* = 27)**	**CC8 (*n* = 30)**	
**DEMOGRAPHIC DATA**			
Age (years, median)	68.05	35.4	**<** **0.001**
**Gender**			
Male	6 (22.2)	13 (43.4)	0.16
Female	21 (77.8)	17 (56.7)	
**Ethnicity**			
Caucasian	22 (81.5)	23 (76.7)	0.75
Non-Caucasian	5 (18.5)	7 (23.3)	
**OCULAR HISTORY**			
History of contact lens wear	8 (29.6)	5 (16.7)	0.35
History of eye trauma	3 (11.1)	4 (13.3)	1
Prior eye surgery	18 (66.7)	5 (16.7)	**<** **0.001**
Previous intraocular lens insertion	12 (44.4)	3 (10.0)	**0.006**
Ocular surface disease^*^	9 (33.3)	5 (16.7)	0.22
Dry eye syndrome	4 (14.8)	4 (13.3)	1
Atopic eye disease	1 (3.7)	1 (3.3)	1
Lid disease^**^	9 (33.3)	6 (20.0)	0.37
Lacrimal system dysfunction^***^	5 (18.5)	5 (16.7)	1
Glaucoma	6 (22.2)	1 (3.3)	**0.05**
Glaucoma drainage device	1 (3.7)	0 (0)	0.47
**HEALTHCARE EXPOSURES**			
Use of topical antibiotic at presentation	15 (55.6)	3 (10.0)	**<** **0.001**
Use of topical antibiotic within the last week preceding presentation	13 (48.5)	5 (16.7)	**0.02**
Use of topical steroids at presentation	11 (40.7)	5 (16.7)	0.08
Use of topical steroids within the last week preceding presentation	10 (37.0)	5 (16.7)	0.13
Known inpatient hospital admission in the last 3 months	4 (14.8)	3 (10.0)	0.7
Admission for eye surgery in the last 3 months^‡^	5 (18.5)	2 (6.7)	0.24
Non-acute clinical care^‡‡^	6 (22.22)	1 (3.3)	**0.05**
Healthcare worker^‡‡‡^	4 (14.8)	5 (16.7)	1

a *Out of 68 isolates included, 66 patient records were reviewed. One patient had two separate MRSA episodes (caused by different clones) and for one patient the record was not available*.

* *includes corneal degenerative disease, bullous keratopathy, exposure keratopathy, dry eye syndrome, atopy, Stevens-Johnson syndrome and toxic epidermal necrolysis*.

** *including blepharitis, trichiasis, floppy eyelid syndrome, ectropion and entropion, and lagophthalmos*.

*** *including dacryocystitis and dacryoadenitis*.

‡ *including day-only procedures and overnight stays*.

‡‡ *includes long-term care residents, nursing home care, chronic ambulatory care*.

‡‡‡ *includes professions in close working contact with patients (e.g., nursing and allied health)*.

*Bold values are represent statistically significant*.

### Distribution of MRSA Lineages Across Different Ocular Niches

Although *S. aureus* causes a wide range of human infections, patterns of association of distinct genotypes have been noted with specific types of infection (29–32). The most well documented example is community-acquired skin and soft-tissue infection (SSTI) in the US, where a large majority of cases are caused by the CC8/USA300 lineage (29, 32). In agreement, ocular SSTIs including mainly orbital and preseptal abscess/cellulitis were found in this study to be caused mainly by CC8 SCC*mec* IV PVL-positive strains, characteristics of the USA300 clone (*p* < 0.001; Table 3). Interestingly, infections of the wet epithelial tissues of the ocular surface were substantially enriched in the CC5 lineage, which was particularly pronounced for infectious keratitis cases (*p* < 0.001; Table 3). Keratitis patients frequently presented with potentially sight-threatening corneal ulcers (85.7%) according to the 1,2,3 rule (23) for categorization of the severity of bacterial keratitis (**Table 5**).

**Table 3:** Distribution of the main ocular MRSA clones across different infections.

**Eye infection**	**Total no. of cases**	**No. of eyes infected by**			**Fisher's test (CC5 vs. CC8)**
		**CC5**	**CC8**	**Others**	
Abscess/Cellulitis	27	1	22	4	**e<0.001**
Keratitis	14	12	1	1	**<0.001**
Conjunctivitis	9	6	2	1	0.1300
Lacrimal system	8	5	2	1	0.2349
Eyelids	4	–	4	–	–
Endopthalmitis	2	2	–	–	–
Miscellaneous	4	2	1	1	–

*Bold values are represent statistically significant*.

### CC5 Strains Preferentially Associated With Infection of the Wet Epithelial Ocular Surface Are Multidrug Resistant

**Figure 2:**
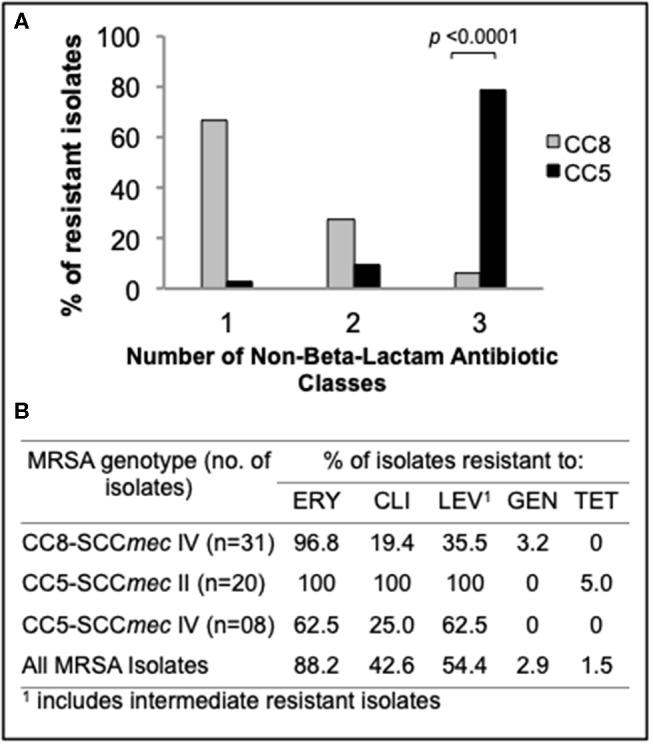
Antimicrobial susceptibility profile of the main ocular MRSA clonal complexes. **(A)** Frequency (%) of CC5 and CC8 strains resistant to additional non-beta-lactam antibiotic classes. **(B)** Resistance rates for common antibiotics representing 5 different classes. Statistical significance was determined using Fisher's exact test.

To compare rates of antibiotic resistance among ocular MRSA of various lineages and from the different ocular sites, we tested the sensitivities of all isolates to a panel of clinically relevant antibiotics using an automated microbiology system (MicroScan WalkAway). Overall, moderate to high rates of resistance to erythromycin (88.2%), levofloxacin (54.4%), and clindamycin (42.6%) was found among this MRSA collection. Resistance to gentamycin (2.9%) and tetracycline (1.5%) was rare (Figure 2B). Stratified analysis by clonal complex showed that CC5 strains are significantly more likely to be multidrug resistant (resistance to ≥3 non-beta-lactam antimicrobial classes) than CC8 strains (78.8 vs. 6.1%; *p* < 0.0001; Figure 2A). Ocular CC5-SCC*mec* II strains, which include isolates resembling the hospital-adapted clone USA100, were all resistant to erythromycin, clindamycin and levofloxacin (Figure 2B). CC5-SCC*mec* IV strains, including isolates with the characteristics of the USA800 clone, were frequently resistant to erythromycin (62.5%), levofloxacin (62.5%), and clindamycin (25.0%). While CC5 strains were frequently resistant to ≥3 non-beta-lactam antibiotics, CC8 isolates were usually resistant to only one antibiotic class in addition to beta-lactams, most often to the macrolide erythromycin (Figures 2A,B).

Because topical fluoroquinolone is widely used for empirical treatment of ocular infection, we assessed the *in vitro* susceptibility of the main ocular MRSA clonal complexes for the most commonly used fluoroquinolones (Table 4). The minimum inhibitory concentrations (MICs) for these agents were determined by reference broth microdilution (24, 25). CC5 isolates were in general highly resistant to the older fluoroquinolones ciprofloxacin, ofloxacin and levofloxacin. All canonical CC5-SCC*mec* II strains were resistant to oxifloxacin, levofloxacin, ofloxacin and ciprofloxacin with MIC_90_ values >256 μg/mL. Among CC5-SCC*mec* IV strains, the MIC_90_ values ranged from 64 μg/mL for moxifloxacin to >256 μg/mL for levofloxacin, ofloxacin and ciprofloxacin (% of non-susceptible = 62.5% for the 3 drugs). Remarkably, many CC5 isolates were also highly resistant to the newer 8-methoxyfluoroquinolone moxifloxacin (MIC_90_ 64 μg/mL, 62.5% non-susceptible for CC5-SCC*mec* IV; MIC_90_ 32μg/mL, 100% non-susceptible for CC5-SCC*mec* II).

**Table 4:** *In vitro* susceptibility profile for topically used fluorquinolones among ocular MRSA isolates according to the clonal group.

**Antimicrobial agent**	**CC8-SCC*****mec*** **IV (*****n*** **=** **31)**		**CC5-SCC*****mec*** **II (*****n*** **=** **20)**		**CC5-SCC*****mec*** **IV (*****n*** **=** **08)**	
	**MIC_**90**_**	**%NS^a^**	**MIC_**90**_**	**%NS^a^**	**MIC_**90**_**	**%NS^a^**
Moxifloxacin	2	35.5	32	100	64	62.5
Levofloxacin	8	35.5	>256	100	>256	62.5
Ofloxacin	16	38.7	>256	100	>256	62.5
Ciprofloxacin	32	42.0	>256	100	>256	62.5

a *includes intermediate resistant isolates*.

## Discussion

The population structure of *S. aureus* isolates from human infections is highly diverse (33). However, only a small proportion of these lineages have become successful MRSA clones that are now widely disseminated in both community and hospital settings (5). These epidemic lineages cause a variety of human infections, with some showing strong tropisms for specific body niches (29–32).

The eye has developed unique mechanisms to protect its delicate and exceptionally important structures against constant external disturbance (15–17). Because of this, we postulated that the uniqueness of this environment could be a driving force shaping the population structure of ocular MRSA infections, in a similar manner as reported for *Streptococcus pneumoniae* (20). To begin testing this hypothesis, we selected a collection of consecutive and non-duplicate MRSA strains prospectively isolated from a variety of eye infections, representing two major and distinct localizations: (i) the ocular surface and (ii) the ocular adnexal soft tissues. We found that the ocular MRSA population was dominated by two major clonal complexes that are also common causes of MRSA infections in other body sites: CC8 (47.0%) and CC5 (41.2%). The distribution of lineages within these clonal complexes followed a pattern of enrichment that was split into the main ocular niches tested, with preseptal and orbital abscess/cellulitis being predominantly associated to CC8, while ocular surface infections were frequently caused by CC5 strains, with a significant enrichment in keratitis (Table 3).

The basis for the discernable tropism of CC5 strains for the ocular surface is unknown, but viewed in light of the findings of others, we can develop a testable model. Strains grouped within CC5, notably the USA100 and USA800, are well established as major causes of healthcare-related infections in the US (5). CC5 strains are often replete with acquired antibiotic resistances (34, 35), and were implicated in the first 12 cases of frank vancomycin resistant *S. aureus* stemming from independent acquisitions of the *van*A gene from *Enterococcus* spp. (36). Despite their hospital association, CC5-MRSA strains also occur in the community, potentially bridged by long-term care facilities. The nasal reservoir of MRSA of long-term care facility residents mirrors the molecular epidemiology of US hospitals, with CC5-related strains being predominant (37). Data from national surveillance programs show that CC5 lineages USA100 and USA800 are now widely disseminated in the community, even among noninstitutionalized individuals with no known risk factors for nasal colonization with these hospital-adapted clones (38). Although CC5 strains are frequently found among carriers in the community, they are only occasionally associated with community-acquired infection (32). In our population, despite the community origins of the patients in which ocular infections occurred, CC5 strains predominated as causes of infection of the wet epithelial surface of the eye. Keratitis in our series was predominantly caused by strains resembling the USA100 lineage, and mostly presented as severe and potentially sigh-threatening infections as determined the “1, 2, 3-Rule” (23). The severity of CC5-caused keratitis points toward the possible existence of virulence factors that could be particularly associated with corneal damage. In addition to carrying a variety of antimicrobial resistance genes, CC5 strains also posses a constellation of virulence genes (35). Of particular interest is the enterotoxin gene cluster (*egc*), which represents a unique group of enterotoxins with superantigen activities, and seems to be particularly enriched among CC5 strains (35), while being completely absent in CC8 strains (36, 39). Epidemiological observations have found an association of *egc*-encoded enterotoxins in the development of corneal ulcer in patients with atopic keratoconjunctivitis (40). Whether this locus is associated with exacerbation of the ocular surface inflammatory response and aggravation of the corneal damage is yet to be fully elucidated.

Empirical use of topical broad-spectrum antibiotics remains the first-line treatment for bacterial keratitis (41) and is commonly initiated with a topical newer fluoroquinolone ophthalmic solution (e.g., moxifloxacin 0.5%) (42). CC5 strains display generally higher fluoroquinolone MICs (non-susceptibility rate for moxifloxacin of 89.3%, MIC_90_ 64 μg/mL) (Table 4). However, CC8-SCC*mec* IV strains associated with ocular infections at other sites were also often resistant to the commonly used fourth-generation fluoroquinolone, moxifloxacin (non-susceptibility rate using systemic breakpoints of 35.5%, MIC_90_ 2 μg/mL). The influence of degrees of resistance on population structure is unclear. In light of its pharmacokinetics on the ocular surface, neither CC5 nor CC8 would be predicted to respond to topical moxifloxacin therapy. Based on pharmacokinetic data for topical moxifloxacin in the cornea of pigmented rabbits (43), we calculated PK/PD (AUC_0−24h_/MIC_90_ ratio) indices for the main MRSA lineages examined in our study (Figure S1). Although the index was higher for CC8 strains compared to CC5, all the indices were far below the PK/PD target that predicts clinical efficacy as determined for systemic infections (44) and also for keratitis treated with fluoroquinolone (45). In addition, a review of the medical records of 14 patients with MRSA keratitis included in this study showed that only 4/14 had prior exposure to a fluoroquinolone (Vigamox, *n* = 2; erythromycin plus ofloxacin, *n* = 1; and Vigamox plus tobramycin, *n* = 1), with no information available for 3 patients (Table 5). Together, the data on prior topical antibiotic use and the calculated PK/PD indices for the ocular MRSA lineages discounts the role of direct fluoroquinolone selection as the driver of the CC5 predominant population structure associated with keratitis.

**Table 5:** Molecular typing and antimicrobial resistances of MRSA keratitis cases.

**Patient**	**Year**	**Sequence type**	**Clonal complex**	**SCC*mec* type**	**PVL**	**Resembles clonal lineage**	**Antimicrobial resistances**	Number of 1, 2, 3 criteria met on presentation^*^	**Topical ATBs at time of culture**
1	2014	ST5	CC5	II	Negative	USA100	OXA, CLIN, ERY, CIP, LEV, OFLX, MOX	3	Vigamox
2	2014	ST105	CC5	II	Negative	SLV of ST5	OXA, CLIN, ERY, CIP, LEV, OFLX, MOX	3	None
3	2014	ST5	CC5	IV	Negative	USA800	OXA	1	UKN
4	2014	ST5	CC5	II	Negative	USA100	OXA, CLIN, ERY, CIP, LEV, OFLX, MOX	0	None
5	2014	ST5	CC5	II	Negative	USA100	OXA, CLIN, ERY, CIP, LEV, OFLX, MOX	2	UKN
6	2015	ST5	CC5	IV	Negative	USA800	OXA, CLIN, ERY, CIP, LEV, OFLX, MOX	2	Polytrim + Erythromycin
7	2015	ST5	CC5	II	Negative	USA100	OXA, CLIN, ERY, CIP, LEV, OFLX, MOX	1	UKN
8	2015	ST5	CC5	II	Negative	USA100	OXA, CLIN, ERY, CIP, LEV, OFLX, MOX	3	None
9	2015	ST96	CC96	NT	Negative	NA	OXA, ERY	3	None
10	2015	ST8	CC8	IV	Positive	USA300	OXA, CLIN, ERY, CIP, LEV, OFLX, MOX	2	None
11	2015	ST105	CC5	II	Negative	SLV of ST5	OXA, CLIN, ERY, CIP, LEV, OFLX, MOX	2	Eythromycin and Ofloxacin
12	2016	ST5	CC5	II	Negative	USA100	OXA, CLIN, ERY, CIP, LEV, OFLX, MOX	0	Vigamox + Tobramycin
13	2016	ST3390	CC5	II	Negative	SLV of ST5	OXA, CLIN, ERY, CIP, LEV, OFLX, MOX	3	Vigamox
14	2016	ST5	CC5	II	Negative	USA100	OXA, CLIN, ERY, CIP, LEV, OFLX, MOX	3	None

* *The “1, 2, 3-Rule,” originally conceived by Vital et al. (23), was formally implemented across MEE on July 1, 2015 as a means of identifying patients at greatest risk of developing sight-threatening complications. It is composed of three simple features on clinical examination, and the presence of any of these warrants the collection of corneal cultures and treatment with topical fortified antibiotics. These rules are: ≥1+ anterior chamber cells; ≥2 mm infiltrate and/or ≥2 satellite lesions; edge of lesion within 3mm of the corneal center*.

Our results are consistent with a report of 30 cases of *S. aureus* keratitis in Japan, that also found CC5 strains to be the predominant cause of MRSA-infected ulcers (46). CC8 isolates were more frequently isolated from the healthy conjunctival sac (46). Together, these findings suggest an enhanced ability of CC5 to endure selective pressures and colonize the cornea and adjacent tissues. Lactoferrin, one the most abundant tear proteins with antimicrobial activity is inhibitory for *S. aureus*, including MRSA, but this activity varies among clinical isolates from different sites of isolation (47, 48). In one study, clinical *S. aureus* isolates causing bloodstream infections (50%) were frequently resistant to lactoferrin concentrations ≥ 20 μM, while isolates from conjunctivitis (33%) and SSTI (13%) were less often resistant (47). Since these infections are caused by distinct MRSA lineages, with CC5 strains being commonly associated to bloodstream infections (49), variations across different genotypes may contribute to increased resistance to this tear antimicrobial compound, especially among CC5. Similarly, the antimicrobial activity of phospholipase A2 (PLA_2_), an enzyme that has been found to be the major tear molecule with bactericidal activity against staphylococci, varies against methicillin-susceptible and -resistant isolates, with MRSA being more resistant to its action following short incubations (50), pointing toward a variance of this enzyme in its ability to kill *S. aureus* according to the genetic background. Further, previous reports have demonstrated that *S. aureus* strains may be differently equipped to bind to and invade ocular tissues *in vitro* and in a rabbit model of conjunctivitis and keratitis (51, 52), suggesting the existence of specific sets of adhesive surface proteins that enhance *S. aureus* ability to bind and invade the ocular tissues in a strain-specific manner.

A recent study of *S. aureus* keratitis in South Florida (53) found both CC5 (40%) and CC8 (37.3%) MRSA among isolates, clearly showing that although CC5 strains are most common, CC8 strains in other circumstances are also capable of causing keratitis. Among our patients, 12 out of 14 (85.7%), were classified as potentially sight-threatening MRSA keratitis cases according to an institution-wide grading system based on the “1, 2, 3” rule (23). For these patients, corneal cultures were unequivocally positive at diagnostic levels for the pathogen. We do not yet know whether the differences between studies stem from levels of disease severity, or other factors such as association with contact lens wear. Larger studies with well-defined criteria for enrollment will be important for determining conditions that favor infection by various pathogenic, antibiotic resistant lineages of *S. aureus*.

In contrast to the wet epithelial surface of the eye, CC8 MRSA strains, typified by the USA300 lineage, predominate as causes of infections of the keratinized epithelium and eye soft tissues. Lineage enrichment has been also reported in patients with bloodstream infection with haematogenous complications (30) infective endocarditis (31) and respiratory tract infections (54). Among our patients, CC8 strains were the predominant causes of preseptal and orbital abscess/cellulitis (22 out of 27 cases), consistent with their known tropism for infecting keratinized epithelium and soft tissues (29, 32). Among the remaining abscess/cellulitis cases, only one was caused by a CC5 isolate and 3 by other lineages (Table 3). In the early 2000s, the USA300 clone appeared in outbreaks of community-acquired SSTIs in otherwise healthy people (55, 56). Because of its ability to rapidly spread through person-to-person contact and readily compete with commensal skin flora (56), the USA300 lineage quickly predominated as the main cause of SSTIs (29, 32), and a significant cause of severe invasive infections (6). USA300 is generally more virulent, causing infections of greater severity and associated with worse outcome compared to other MRSA clones (57, 58). The aggressiveness of USA300 type strains is thought to be associated to the carriage of a variety of virulence factors, including the pore-forming toxin PVL, which is predominantly found in CC8 strains (59), and has been linked to the ability of this strain to cause necrotizing infections (60).

Orbital and preseptal abscesses are SSTIs commonly caused by *Staphylococcus aureus* (61, 62), and MRSA rates appear to be rising (63). Severe cases have been reported, including orbital and periorbital necrotizing fasciitis (64–66), necrotizing conjunctivitis with orbital invasion (12) and orbital cellulitis with bilateral involvement that progressed to blindness (14). A prospective study (2012–2015) of children and adolescents presenting with staphylococcal periorbital and orbital cellulitis in Houston found that most of the *S. aureus* isolates in their population were methicillin-resistant (67%) and were genetically related to the USA300 clone (78%) (67). Similarly, in a series of 11 patients presenting with culture-positive MRSA infections of the eye and orbit in San Francisco, most (82%) were caused by USA300 (13). Preseptal and orbital abscess/cellulitis were among the most common manifestations in these cases, some presenting with extensive necrosis of the eyelid and orbital tissues.

Collectively, these results are consistent with various MRSA lineages being enriched for properties that enhance their ability to resist defenses and competitive pressures, and colonize and infect either the wet epithelial surface or the ocular adnexal tissues. We propose that many of the features that have allowed CC5-type MRSA to adapt and be transmitted in the hospital environment, and readily acquire antibiotic resistances, endows them with properties that enhance their ability to resist robust ocular surface defenses and infect the cornea. In a comparative analysis of the genomes of the CC5 strains that had acquired vancomycin resistance from enterococci, we identified a constellation of traits with the *S. aureus* pathogenicity island as likely involved with their ability to persist in a mixed infection and acquire resistances by horizontal gene transfer (36). These same traits may well enhance their survival despite the defenses of the wet epithelial surface. In contrast, CC8-SCC*mec* IV PVL-positive strains are already well known to have features that enhance their ability to colonize the skin and compete effectively for that niche with coagulase negative strains, including the ACME locus (68). We believe these features account for their association with infection of the keratinized surfaces of the ocular adnexa. Direct testing of isogenic mutants will be required to identify the major contributors to pathogenesis of various anatomical sites of the eye by MRSA, and to develop new approaches for mitigating that common threat. It is important to note that these findings represent the population structure of a relatively small ocular MRSA population isolated at the Massachusetts Eye and Ear, and validation of our results using a larger bacterial collection and isolates from other locations would be warranted.

## Data Availability Statement

The datasets generated for this study are available on request to the corresponding author.

## Author Contributions

PB, LU, JC, and MG contributed conception and design of the study and wrote sections of the manuscript. PB and LU organized the database and performed the statistical analysis. PB wrote the first draft of the manuscript. All authors contributed to manuscript revision, read and approved the submitted version.

## Conflict of Interest

The authors declare that the research was conducted in the absence of any commercial or financial relationships that could be construed as a potential conflict of interest.

## Supplementary Material

Figure S1 PK/PD (AUC_0−24h_/MIC_90_ ratio) indices for the main MRSA clones isolated in our study. AUC_0−24h_ data was derived from pharmacokinetic studies of topical moxifloxacin in the cornea of pigmented rabbits (43). Dashed line indicates the PK/PD target that predicts clinical efficacy in keratitis patients treated with topical fluoroquinolone (45).
