# Supplementary figures and images for: Hospital-Associated Multidrug-Resistant MRSA Lineages Are Trophic to the Ocular Surface and Cause Severe Microbial Keratitis
Source: Front Public Health. 2020 Jun 3;8:204. doi: 10.3389/fpubh.2020.00204 (PMC7283494; doi:10.3389/fpubh.2020.00204)

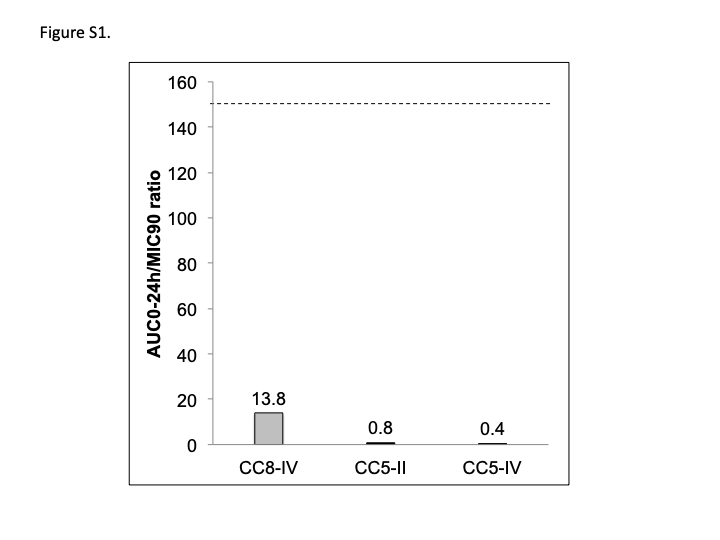

Supplement: Figure S1 — PK/PD (AUC0−24h/MIC90 ratio) indices for the main MRSA clones isolated in our study. AUC0−24h data was derived from pharmacokinetic studies of topical moxifloxacin in the cornea of pigmented rabbits (43). Dashed line indicates the PK/PD target that predicts clinical efficacy in keratitis patients treated with topical fluoroquinolone (45). [file Image_1.TIFF]
